# Repeated treatment with bone marrow cell secretory products maintains long-term renoprotection in experimental chronic kidney disease: a placebo-controlled trial

**DOI:** 10.1186/s40697-015-0082-5

**Published:** 2015-11-14

**Authors:** Darren A. Yuen, David M. Kepecs, Yanling Zhang, Suzanne Advani, Kerri Thai, Kim A. Connelly, Richard E. Gilbert

**Affiliations:** Keenan Research Centre for Biomedical Science of St. Michael’s Hospital, 209 Victoria Street, Toronto, Ontario M5B 1T8 Canada; Division of Endocrinology, Department of Medicine, Keenan Research Centre for Biomedical Science of St. Michael’s Hospital, 209 Victoria Street, Room 508, Toronto, ON M5B 1T8 Canada

**Keywords:** Chronic kidney disease, Cell therapy, Early outgrowth cell, Conditioned medium

## Abstract

**Background:**

Bone marrow-derived early outgrowth cells (EOCs) secrete soluble factors that exert potent renoprotective effects, such that infusion of their conditioned medium recapitulates the affects of the cells themselves.

**Objectives:**

The objective of this study is to test whether the protective effect of conditioned medium infusion wanes with time and whether tachyphylaxis occurs with repeated administration.

**Design:**

This is a placebo-controlled animal study.

**Setting:**

The study was conducted at St. Michael’s Hospital, Toronto, Ontario, Canada.

**Subjects:**

Fischer 344 (F344) rats were used in this study.

**Measurements:**

The following were measured: (1) urinary protein:creatinine ratio, (2) glomerular filtration rate, (3) systolic blood pressure, (4) body weight, (5) glomerular endothelial cell density, and (6) glomerular and tubulointerstitial type IV collagen deposition.

**Methods:**

Subtotally nephrectomized F344 rats, a model of progressive chronic kidney disease, were randomized 4 weeks post-surgery to receive thrice-weekly intravenous injections of concentrated EOC-conditioned medium (EOC CM) or unconditioned medium (UCM) over 2 weeks. Three animal groups were studied, according to whether they were administered conditioned medium: once (Initial Therapy Only group), twice (Repeat Therapy group), or not at all (No Therapy group).

**Results:**

Following initial therapy, EOC CM-treated animals excreted less urinary protein, a marker of renal injury, than their UCM-treated counterparts. At 10 weeks post-subtotal nephrectomy, however, mean urinary protein excretion in conditioned medium-treated animals was fourfold greater than at the completion of the initial treatment course. At this time point, conditioned medium-treated animals were randomized to receive a second course of either conditioned medium (Repeat Therapy group) or unconditioned medium (Initial Therapy Only group). At study end (14 weeks post-subtotal nephrectomy), Repeat Therapy animals demonstrated higher glomerular filtration rate, less proteinuria, preserved renal microvasculature, and diminished fibrosis when compared with the No Therapy group. Initial Therapy Only animals exhibited an intermediate effect.

**Limitations:**

Testing the effect of EOC-conditioned medium in a single model of chronic kidney disease (CKD) has limitations.

**Conclusions:**

These findings suggest that early outgrowth cell-derived factors, while renoprotective, have a limited duration of action. Repeated administration of these factors, however, is able to extend the duration of efficacy and attenuate the progression of experimental chronic kidney disease.

## Abrégé

**Contexte:**

Les cellules à croissance précoce (CCP) provenant de la moelle osseuse sécrètent des facteurs solubles qui procurent des effets néphroprotecteurs très efficaces. Une simple infusion de milieu de culture conditionné par des CCP permet d’instaurer des effets protecteurs dans les cellules rénales.

**But de l’étude:**

Vérifier si l’effet néphroprotecteur d’une infusion de milieu conditionné décline dans le temps et déterminer si l’administration répétée engendre une tachyphylaxie.

**Type d’étude:**

Essai contrôlé par placebo sur modèles animaux

**Établissement:**

Hôpital St. Micheal’s de Toronto, ON, Canada

**Sujets:**

Rats Fisher (F344)

**Mesures:**

(1) le ratio urinaire « protéines/créatinine » (2) le débit de filtration glomérulaire (3) la pression systolique (4) le poids corporel (5) la densité des cellules endothéliales glomérulaires et (6) le dépôt tubulo-interstitiel et glomérulaire de collagène de type IV.

**Méthode:**

Quatre semaines suivant la chirurgie, deux groupes expérimentaux ont été formés par randomisation de rats F344 partiellement néphrectomisés (un modèle utilisé pour l’étude de l’insuffisance rénale chronique et dégénérative). Durant 2 semaines, les animaux recevaient 3 injections intraveineuses par semaine d'un concentré de milieu conditionné par cellules à croissance précoce (MC-CCP) ou d'un concentré de milieu non conditionné (MNC). Les animaux du groupe MC-CCP ont, plus tard au cours de l’étude, été divisés en 2 sous-groupes. L’un d’eux a reçu une deuxième série d’injections de MC-CCP et l’autre, de MNC. Ainsi, 3 groupes ont été étudiés, selon qu’ils ont été traités au MC-CCP une seule fois (groupe « Traitement Initial Seulement »), deux fois (groupe « Traitement Répété ») ou en aucun temps (groupe « Non Traité »).

**Résultats:**

Dès le traitement initial, une diminution de l’excrétion urinaire de protéines (un indicateur de lésions aux reins) a été observée chez les sujets du groupe MC-CCP par rapport aux niveaux de leurs homologues du groupe MNC. Par contre, la moyenne d’excrétion urinaire de protéines chez les sujets du groupe MC-CCP avait tout de même quadruplé, 10 semaines suivant la chirurgie, par rapport aux taux mesurés après la première ronde de traitements. À ce stade de l’étude, les sujets du groupe MC-CCP ont été divisés et randomisés en deux sous-groupes à qui une deuxième ronde de traitements, soit par MC-CCP (groupe « Traitement Répété »), soit par MNC (groupe « Traitement Initial Seulement ») a été administrée. Au terme de l’étude (14 semaines suivant la chirurgie), les animaux du groupe « Traitement Répété » ont démontré un débit de filtration glomérulaire supérieur, une diminution de la protéinurie, une préservation de la microvasculature rénale et une réduction de la fibrose, lorsque comparés au groupe non traité. Les animaux du groupe « Traitement Initial Seulement » ont montré des résultats intermédiaires.

**Limitations:**

L’effet d’un traitement avec MC-CCP n’a été testé que dans un seul modèle expérimental d’insuffisance rénale chronique.

**Conclusions:**

Ces constatations suggèrent que, tout néphroprotecteurs qu’ils soient, les facteurs solubles dérivant de la culture de cellules à croissance précoce provenant de moelle osseuse ont une action de courte durée. En revanche, il apparait qu’une administration répétée de ces facteurs permet d’en prolonger l’action tout en freinant la progression de l’insuffisance rénale chronique dans les modèles expérimentaux.

## Background

Despite inducing improvements in organ structure and function, the mechanisms by which bone marrow-derived cell therapy affects these changes remain uncertain. Previously thought to engraft and transdifferentiate [1–4], more recent opinion favors a paracrine mechanism of action whereby the administered cells secrete a range of factors that initiate endogenous regenerative programs by cells in their immediate proximity [5].

The paucity or absence of the administered cells at sites of injury, however, has led several studies to examine whether an endocrine mode of action might also apply in certain settings. In these studies, the systemic administration of conditioned medium, rather than the cells themselves, was shown to be effective. One of the first reports documenting the protective activity of such secreted factors demonstrated that intraperitoneal delivery of conditioned medium from marrow stromal cells attenuated cisplatin-induced acute kidney injury [6]. Similarly, intravenously administered conditioned medium derived from embryonic mesenchymal stem cells [7] was able to attenuate kidney injury in the subtotally nephrectomized rat model of chronic kidney disease. We have similarly shown that conditioned medium from early outgrowth cells, an alternate bone marrow-derived mononuclear cell population, can attenuate injury progression in the subtotally nephrectomized rat, attenuating fibrosis and preserving microvasculature in the injured kidney [8].

Cell-free conditioned medium has the potential to mitigate the concerns associated with cell therapy, such as the neoplastic transformation of autologous cells [9] or the immune considerations that accompany allogeneic cell administration. However, unlike cells that would presumably continue to secrete their reparative factors while they remained viable, the durability of conditioned medium is likely more limited. To date, however, no studies have specifically addressed this issue. Following our recent description of the pro-angiogenic effects of EOC-derived conditioned medium, and its ability to attenuate experimental chronic kidney disease progression [8, 10], the aims of the present study were twofold. We firstly sought to examine the duration of EOC-derived conditioned medium’s effectiveness. Secondly, if the effects were noted to wane with time, we also sought to determine whether repeated administration would be beneficial or instead show tachyphylaxis. Here, we demonstrate that the renoprotective effect of EOC-conditioned medium treatment does, in fact, wane 4 weeks after injection. We further show that retreatment leads to persistent protection, being associated with improved renal function, reduced fibrosis, and less microvascular injury.

## Methods

### Conditioned medium

Conditioned medium was generated as previously described [8]. In brief, bone marrow cells were flushed from the femora and tibia of 4-week-old male Fischer F344 rats and plated in endothelial cell growth medium (EGM-2, Lonza, Walkersville, MD) on human fibronectin-coated tissue culture flasks and incubated at 37 °C with 5 % CO_2_ for 10 days to give rise to EOCs. EGM-2 consists of serum-free endothelial basal medium-2 (EBM-2, Lonza) supplemented with growth factors such as vascular endothelial growth factor, epithelial growth factor, and fetal bovine serum. After repeatedly washing the EOCs with phosphate-buffered saline to remove any residual serum, subconfluent cells were incubated with serum-free EBM-2 for 24 h to collect EOC-released factors [8]. Culture supernatant was then collected and the resulting cell-free, EOC-conditioned medium (EOC CM) was concentrated tenfold using a >10-kDa cutoff centrifuge filtration column (Millipore, Billerica, MA), followed by filtration through a 0.45-μm filter (Millipore). Unconditioned medium (UCM) consisting of serum-free endothelial basal medium-2 not incubated with cells was similarly concentrated. Half-milliliter aliquots of concentrated EOC CM and UCM were stored at −80 °C until administered as thrice-weekly tail vein injections.

### Animal model

Studies were performed in subtotally nephrectomized rats, a model of non-immune progressive kidney disease that like chronic kidney disease in humans is characterized by proteinuria, declining glomerular filtration rate, and hypertension. Twenty-nine, 8-week-old F344 rats underwent one-step subtotal nephrectomy (SNX) according to previously published protocols [8, 11]. All animal studies were approved by the St. Michael’s Hospital Animal Ethics Committee (ACC862) and followed the guidelines for animal use and care as described by the Canadian Council on Animal Care.

### Study design

Four weeks following SNX, rats were randomized to receive unconditioned medium (No Therapy group, *n* = 14) or EOC-conditioned medium (Therapy group, *n* = 15). After an initial 2-week set of thrice-weekly injections of EOC-conditioned medium, Therapy group animals were then randomly assigned to receive either a repeat 2-week course of thrice-weekly conditioned or unconditioned medium injections. Repeat administration (of either conditioned medium or unconditioned medium) to all Therapy group animals was mandated by the study protocol if mean urinary protein excretion rose fourfold relative to the level following completion of the initial treatment course. This cutoff for repeat therapy was chosen (1) to establish when the effect of initial EOC-conditioned medium treatment wanes and (2) to define a threshold for repeat treatment that was clinically relevant. Given the large variability typically seen in urinary protein excretion rates in the subtotal nephrectomy model [8], we reasoned that using a lower threshold might trigger repeat treatment at a time when the effects of initial EOC medium treatment might not have clearly waned.

**Fig. 1:**
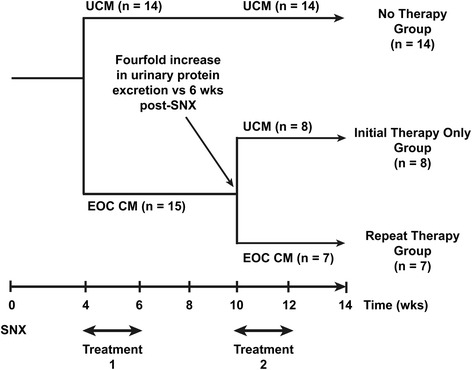
Study design. *UCM* unconditioned medium, *EOC CM* early outgrowth cell-conditioned medium, *SNX* subtotal nephrectomy, *wks* weeks

No Therapy group animals received the initial course of thrice-weekly UCM injections beginning 4 weeks post-SNX and another 2 weeks of unconditioned medium injections when the Therapy groups received their second course of CM. Animals were then monitored for four additional weeks. Accordingly, three groups of animals were studied: a No Therapy group (UCM + UCM, *n* = 14), an Initial Therapy Only group (EOC CM + UCM, *n* = 8), and a Repeat Therapy group (EOC CM + EOC CM, *n* = 7) (Fig. 1).

### Outcome measurements

Systolic blood pressure was measured using a tail-cuff plethysmograph (ADInstruments, Colorado Springs, CO) [8, 11, 12]. Urinary protein excretion was determined as protein:creatinine ratios measured on spot urine collected before surgery and 4, 6, 8, 10, 12, and 14 weeks after. Glomerular filtration rate (GFR) was measured just prior to termination using a modified FITC-inulin plasma clearance method [8].

To assess structure, the kidneys, excised at termination, were immersion fixed in 10 % neutral buffered formalin. Glomerular endothelial cell density, glomerulosclerosis, and tubulointerstitial fibrosis were assessed on JG-12 or type IV collagen immunostained formalin-fixed sections, respectively, as previously described [8]. Analyses of kidney structure were performed in a masked fashion using computer-assisted image analysis, as described previously [12].

### Statistical analysis

All data are shown as mean ± SEM unless otherwise stated. Differences between groups were analyzed by ANOVA with a post hoc Tukey’s test. A change was considered statistically significant if *p* < 0.05.

## Results

### Repeated therapy is required to preserve renal function in the subtotally nephrectomized rat

Whereas proteinuria rose progressively in the No Therapy group, reaching 1740 ± 468 mg/mmol at 10 weeks post-SNX and 2674 ± 602 mg/mmol at study end (14 weeks post-SNX), animals that had received an initial course of EOC CM had an attenuated rise in proteinuria, increasing from 210 ± 62 mg/mmol following completion of the initial EOC CM treatment at 6 weeks post-SNX to 881 ± 138 mg/mmol at 10 weeks post-SNX. At this 10-week post-SNX mark, having experienced more than a fourfold increase in mean urinary protein excretion following completion of initial therapy, EOC CM-treated animals had reached the threshold for retreatment. Rats that had initially received EOC CM were, accordingly, randomized to receive either another course of EOC CM (Repeat Therapy group) or unconditioned medium (Initial Therapy Only group). Of the eight animals randomized to Initial Therapy Only, the mean rise in urinary protein excretion between 6 and 10 weeks post-SNX was 5.6 ± 1.7-fold. Of the seven animals randomized to Repeat Therapy, the mean rise in urinary protein excretion was 7.4 ± 1.6-fold. After this second treatment course, although proteinuria continued to rise in both groups, they did so at different rates, so that by 14 weeks post-SNX, urinary protein excretion was lower in the Repeat Therapy group compared with that in the No Therapy group, with the Initial Therapy Only group showing an intermediate effect (Table 1).

**Table 1:** Renal function at 14 weeks post-surgery

	No therapy	Initial therapy only	Repeated therapy
*N*	14	8	7
Body weight (g)	194 ± 3	195 ± 4	190 ± 5
Glomerular filtration Rate (μL/min/g body weight)	1.35 ± 0.20	1.83 ± 0.10	2.14 ± 0.20*
Urine protein: creatinine (mg/mmol)	2674 ± 602	2536 ± 471	1912 ± 311
Systolic blood pressure (mmHg)	178 ± 20	176 ± 8	167 ± 9

*p < 0.05 vs. No Therapy Group

Glomerular filtration rate, measured at the end of the study, demonstrated similar findings, with the Repeat Therapy group being significantly protected against a fall in GFR compared with the No Therapy group and the Initial Therapy Only group showing an intermediate effect (Table 1). Blood pressure, on the other hand, was similar among all groups (Table 1).

### Kidney structure

**Fig. 2:**
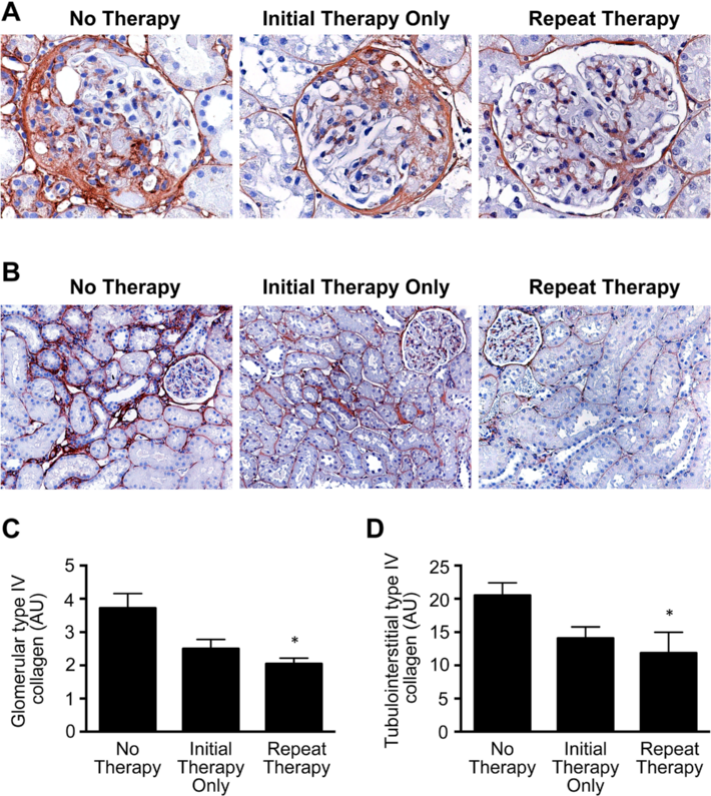
Repeated EOC CM injections lead to continued protection against CKD-associated renal fibrosis. Kidney sections were immunolabeled for collagen IV. **a** Representative glomerular images. Original magnification ×400. **b** Representative tubulointerstitial images. Original magnification ×160. **c** Quantification of glomerular type IV collagen positivity. Thirty randomly selected glomeruli cut through the macula densa were sampled per kidney section (*n* = 11, No Therapy; *n* = 8, Initial Therapy Only; *n* = 7, Repeat Therapy). **d** Quantification of tubulointerstitial type IV collagen positivity. Ten randomly selected, non-overlapping ×20 fields were sampled per kidney section (*n* = 11, No Therapy; *n* = 8, Initial Therapy Only; *n* = 7, Repeat Therapy). *p < 0.05 vs. SNX animals receiving No Therapy. *AU* arbitrary units

Animals that had undergone SNX all showed substantial glomerulosclerosis that was attenuated in rats that received two courses of EOC CM (Repeat Therapy group) when compared with those that had only received unconditioned medium (No Therapy group). Consistent with having received only one course of EOC CM treatment, the Initial Therapy Only animals that had received an initial course of EOC CM followed by unconditioned medium showed levels of glomerulosclerosis that were intermediate between those of the Repeat Therapy and No Therapy groups (Fig. 2). In addition to glomerulosclerosis, SNX rats also developed extensive interstitial fibrosis. When compared with animals that received unconditioned medium only, the extent of fibrosis was reduced in the Repeat Therapy group with the Initial Therapy Only group showing, once again, an intermediate response (Fig. 2).

**Fig. 3:**
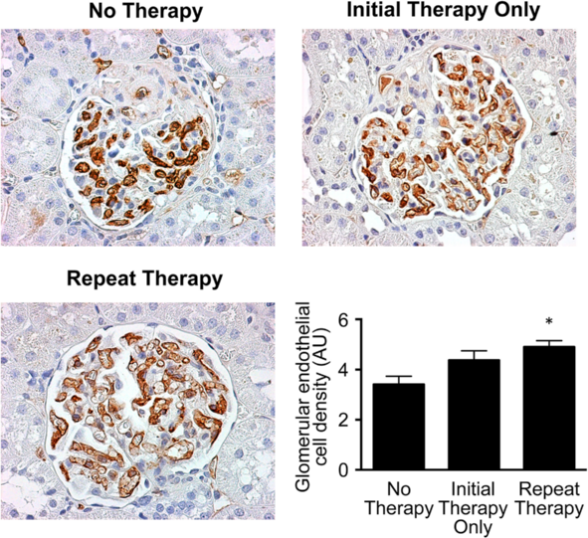
Repeated EOC CM injections lead to continued protection against CKD-associated glomerular capillary rarefaction. Kidney sections were immunolabelled with JG-12 antibody, which binds to aminopeptidase P, a glomerular endothelial antigen. Representative images are presented. Original magnification ×400. Quantification is presented in the *lower right corner*, following sampling of 30 randomly selected glomeruli cut through the macula densa per kidney section (*n* = 11, No Therapy; *n* = 8, Initial Therapy Only; *n* = 7, Repeat Therapy). *p < 0.05 vs. SNX animals receiving No Therapy. *AU* arbitrary units

As glomerulosclerosis is associated with progressive capillary loss in the glomerulus, we next examined the effects of EOC CM therapy on glomerular capillary density following subtotal nephrectomy. As expected, SNX rats that received only unconditioned medium (No Therapy group) developed significant glomerular capillary loss. Consistent with its renoprotective properties, rats receiving EOC CM once (Initial Therapy Only group) were partially protected against this glomerular capillary loss, while rats receiving EOC CM twice (Repeat Therapy group) were protected to an even greater degree (Fig. 3).

## Discussion

Cell therapy holds substantial promise as a new therapy for many chronic diseases, including CKD. In prior work, we demonstrated that a single intravenous infusion of bone marrow-derived early EOCs exerts potent renoprotective effects in models of non-diabetic and diabetic CKD [11, 13]. However, clinical translation of EOC infusions as a treatment for CKD has been hampered by major limitations, including disease-induced dysfunction of autologous cells [10, 14–18], induction of immune responses to allogeneic cells, and the potential for uncontrolled proliferation of these immature cells following injection [9, 19].

One possible approach to avoid such problems is to utilize the secretory products of these cells rather than the cells themselves, thereby obviating the concerns associated with cell administration [8]. However, to be effective, the durability and potential for repeated administration of such factors needs to be ascertained. Here, we show for the first time that while a single course of EOC CM is able to improve renal structure and function in the short term, its renoprotective effects wane with time. Repeated dosing did, however, provide for prolonged structural and functional preservation. In particular, repeated therapy leads to significant preservation of glomerular filtration rate, a key finding that, unlike surrogate outcomes such as urinary protein excretion, is recognized as a valid and critical study outcome [20]. Taken together, these findings suggest that while cell-free conditioned medium may be a viable alternative to cell infusion, the need for its repeated administration seems likely.

Following on from the wealth of studies in cardiovascular disease and acute kidney injury, studies have now begun to assess the effects of cell-based therapies in animal models of chronic kidney disease [11, 13, 14, 21, 22]. Translation to the human setting has, however, been marred by a case report of neoplastic transformation in a recipient of autologous G-CSF-mobilized blood marrow-derived “stem cells” [9]. This report raised serious concerns regarding the fate of infused bone marrow-derived cells, highlighting our relatively limited understanding of the behavior of these cells following in vivo injection [19]. A further concern has been the source of cells for injection. While the use of autologous cells would avoid potential cytotoxic alloimmune responses, recent reports suggest that both diabetes and CKD can reduce the number and the reparative activity of circulating early outgrowth cells [10, 16–18, 23–28], making an autologous cell infusion strategy less attractive. In this context, the results of our study, demonstrating the beneficial effects of repeated administration of a cell-free product, strengthen the case for a novel treatment strategy that bypasses these major safety and efficacy concerns.

In the current study, we tested the beneficial effects of EOC CM retreatment in the SNX rat because this model mimics many features of progressive non-diabetic human CKD, including final common injury pathways such as capillary loss and fibrosis that are activated in virtually all forms of chronic renal injury [29]. However, other processes, such as inflammation- and toxin-induced cell injury, may play significant roles in the pathogenesis of specific forms of CKD. Thus, while a repeat EOC CM treatment strategy protects against fibrosis and microvascular injury in the SNX rat, leading to improvements in renal function, it is important to note that the benefits of EOC CM may not extend to all types of CKD. Future studies will be required to test the potential benefits of EOC CM administration in these other types of chronic renal injury.

## Conclusions

Our findings provide the first evidence supporting the efficacy of repeated administration of EOC conditioned medium for the treatment of chronic kidney disease. In particular, we demonstrate that repeated treatment with EOC-conditioned medium can protect the renal microvasculature, even in the face of ongoing renal injury. These data, in the context of prior work demonstrating the potent renoprotective effects of EOC-derived soluble factors, provide a strong rationale for clinical trials of these factors as a novel therapy for CKD.
